# Functional Outcome of High Tibial Osteotomy and Type-II Undenatured Collagen in Active Middle-Aged Adults in Early Knee Osteoarthritis: A Prospective Comparative Study

**DOI:** 10.7759/cureus.94616

**Published:** 2025-10-15

**Authors:** Yogesh Singh Parihar, Rajveer S Bajoria, Dharam Panthi, Vikas Singhal

**Affiliations:** 1 Department of Orthopaedics and Traumatology, Gajra Raja Medical College, Gwalior, Gwalior, IND; 2 Department of Orthopaedics and Traumatology, Gandhi Medical College, Bhopal, IND

**Keywords:** high tibial osteotomy, knee osteo-arthritis, total knee joint replacement (tka), undenatured type-ii collagen, varus deformity

## Abstract

Knee osteoarthritis (OA), commonly referred to as degenerative joint disease, is characterized by the progressive degradation and loss of articular cartilage. This chronic, debilitating condition increasingly affects not only the elderly but also younger individuals. The medial compartment of the knee is more frequently involved than the lateral, often resulting in a varus deformity. Initial management typically involves conservative measures, including weight management, physiotherapy, and pharmacological therapy. Common medications include paracetamol and non-steroidal anti-inflammatory drugs (NSAIDs). However, concerns regarding long-term safety, particularly in patients with multiple comorbidities, have limited their prolonged use. Consequently, non-pharmacological agents such as undenatured type-II collagen have emerged as promising alternatives. When conservative management fails, surgical intervention becomes necessary. High tibial osteotomy (HTO) is a well-established joint-preserving procedure for medial unicompartmental knee OA. By shifting the mechanical axis toward the lateral compartment, HTO offloads the diseased medial compartment, thereby alleviating pain, improving function, and potentially delaying the need for total knee arthroplasty.

## Introduction

Knee osteoarthritis (OA), also known as degenerative joint disease, is a chronic and progressive condition that results in significant functional disability. Its incidence is increasing, notably among younger and more active populations [1]. Clinically, OA of the knee presents with gradually worsening joint pain, typically aggravated by activity, along with stiffness, swelling, discomfort following prolonged sitting, and functional decline over time.

Initial management typically involves conservative treatment, including lifestyle modification, physiotherapy, and pharmacologic therapy. However, when non-operative measures fail, surgical intervention may become necessary [1].

OA frequently affects the medial compartment of the knee, often leading to a varus deformity, which further exacerbates disease progression by medializing the weight-bearing axis. In such cases, high tibial osteotomy (HTO) has been established as an effective joint-preserving procedure. HTO aims to correct malalignment and relieve symptoms by shifting the mechanical axis toward the relatively preserved lateral compartment, thereby partially unloading the medial compartment [2].

The primary goals of HTO are twofold: (i) to alleviate knee pain by redistributing joint loads in varus-aligned knees, and (ii) to delay or prevent the need for total knee arthroplasty by halting or slowing the progression of medial compartment degeneration. This is achieved through a controlled overcorrection of the mechanical axis, typically to 6°-10° of valgus alignment [2]. Various HTO techniques have been described, including open-wedge, closed-wedge, dome, and "en chevron" osteotomies, with open- and closed-wedge methods being the most commonly utilized. Although the popularity of HTO has declined in recent years due to advancements in knee arthroplasty, careful patient selection, precise preoperative planning, and appropriate surgical technique remain essential for achieving optimal clinical outcomes [3].

In addition to surgical options, several conservative treatments are available for managing early OA, encompassing both pharmacological and non-pharmacological strategies. Common medications include paracetamol and non-steroidal anti-inflammatory drugs (NSAIDs) [4]. However, long-term use of these agents is often limited by safety concerns, particularly in patients with comorbidities [5]. As such, nutritional supplements and nutraceuticals, notably collagen-based therapies, have gained increasing attention.

Native (undenatured) and hydrolyzed collagen are two extensively studied forms. Undenatured type-II collagen (UC-II) functions via an immune-mediated mechanism, promoting oral tolerance by modulating the immune response to prevent joint inflammation and tissue damage. In contrast, hydrolyzed collagen may deliver bioactive peptides that penetrate cartilage and contribute to chondroprotective effects [6].

The primary objective of this study was to evaluate the change in Knee Society Score (KSS) at nine months following medial open-wedge HTO compared with conservative management using NSAIDs and UC-II in middle-aged adults (40-60 years) with Kellgren-Lawrence (K-L) grade I-III medial compartment OA of the knee. The secondary objectives were to: assess improvement in pain using the visual analogue scale (VAS) at three, six, and nine months, evaluate radiographic correction of the mechanical axis following HTO, and document any treatment-related adverse events or complications occurring during follow-up.

Clinical significance

Understanding the short-term functional and pain outcomes of HTO compared to conservative UC-II-based therapy may help guide treatment selection and joint-preserving strategies in middle-aged patients with early medial knee OA.

## Materials and methods

This study is a non-randomized, prospective, clinical study conducted at Jay Arogya Group of Hospitals (JAH), Gaja Raja Medical College, Gwalior, Madhya Pradesh, India, from October 2022 to May 2024. Eligible patients who met the inclusion criteria were offered two treatment options: high tibial osteotomy (HTO) or conservative management with UC-II and NSAIDs, and were allocated based on their informed preference. Because allocation was not randomized, the study is best classified as a non-randomized prospective cohort.

The study was approved by the Institutional Ethics Committee, Gajra Raja Medical College, Gwalior (approval number: 68/IEC-GRMC/2022) and was reported in accordance with the STROBE (Strengthening the Reporting of Observational Studies in Epidemiology) guidelines. Elements from the TREND (Transparent Reporting of Evaluations with Nonrandomized Designs) statement were also considered to enhance transparency in reporting non-randomized interventions. The included patients were classified in grades I to III on the Kellgren-Lawrence grading scale after prior well-informed written consent. All procedures were performed in accordance with the ethical standards of the institutional and national research committee and with the 1964 Helsinki declaration and its later amendments. Written informed consent was obtained from all participants prior to inclusion in the study.

Eligibility criteria

The inclusion criteria were pain and disability resulting from OA that interfered with high-demand employment or recreation, age 40-60 years, Grade I to III Knee OA on Kellgren-Lawrence grading scale, and the patient giving consent for examination and follow-up. Exclusion criteria were patients with secondary OA, patients who have received steroid injections within the past six months, patients with more than 20 degrees of correction needed in surgery, patients with hemoglobin levels less than 10 gm/dl, patients with tumours, and metabolic diseases of bone.

Sample size calculation

The sample size was calculated based on an assumed prevalence of knee OA of 3.62%, with 5% level of significance and 5% absolute error, using the formula $n = \frac{(Z_{\alpha/2})^2 \, P Q}{d^2}$, where n is the required sample size, Z_α/2_ is the standard normal variate corresponding to the desired confidence level (for 95% confidence, Z_α/2_=1.96), P is the estimated prevalence or proportion of the condition (e.g., prevalence of knee OA = 3.62%), Q=1−P is the complement of the prevalence, and d is the allowable margin of error or absolute precision (commonly 5%, i.e., 0.05). This yielded a sample size of 54, and accordingly, 27 patients were included in each group.

Eligible patients were counseled regarding both surgical and conservative treatment options, and allocation to either group was based on patient preference after informed consent. This method of allocation may introduce selection bias and potential confounding by indication.

Study procedure

During the first visit, a detailed physical examination, baseline investigations including complete blood count (CBC), erythrocyte sedimentation rate (ESR), C-reactive protein (CRP), liver function tests (LFT), kidney function tests (KFT), and rheumatoid factor were performed in all patients to exclude systemic inflammatory disease, metabolic or organ dysfunction, and to confirm suitability for surgical or conservative management. These investigations were used for screening only and are not reported as outcome measures. In addition, conventional standing anteroposterior (AP) and lateral knee radiographs with bilateral lower limb scannogram were taken before the initiation of investigational product UC-II with traditional NSAIDs or before planning for high tibial osteotomy.

The KSS (1989 version) and VAS were used to assess function and pain, respectively. Outcome assessments were conducted by clinicians who were aware of patient treatment allocation; thus, the study was not blinded. All patients were divided into two groups of 27 each, according to their consent for the intervention of surgery. Group I underwent HTO for knee OA, and Group II received conservative medical treatment.

Group I patients were selected for HTO surgery after preoperative evaluation for OT and were admitted after fitness evaluation from an anesthetist. The preoperative varus angle was calculated, and the correction needed was also calculated using the Miniaci method. In HTO, a skin incision was made on the medial aspect of the proximal tibia, and a longitudinal incision starts just below the joint line between the medial border of the patellar ligament and the posterior margin of the tibia. Subcutaneous tissue was dissected, and the pes anserinus was retracted posteriorly, which exposed the medial collateral ligament. The long fibres of the superficial medial collateral ligament were then detached until the postero-medial cortex of the proximal tibia was exposed. The leg was then placed in full extension, and the knee joint was placed in exact AP view under fluoroscopy. A 2 mm K-wire was passed starting from the medial cortex about 4 cms below the joint line to proximally towards the lateral cortex about 1 cm below the joint line. The second K-wire was passed parallel and anterior to the first one. Using a saw, an osteotomy cut was made along the K-wires, leaving 1 cm of lateral cortex intact. The osteotomy site was opened slowly with valgus stress. After opening the osteotomy site, the HTO plate, along with the attached metal block, was inserted and locked with locking screws. Plate position was checked radiographically with X-ray.

Both groups received NSAIDs (aceclofenac 100 mg twice daily) as part of standard symptomatic management, and the HTO group received standard postoperative analgesic and rehabilitation care. Rehabilitation included (i) static quadriceps and ankle pumping exercises on the day of surgery, (ii) non-weight-bearing walking for six weeks, (iii) partial weight bearing for 6 to 12 weeks, (iv) complete weight bearing after 12 weeks, and (v) follow-up evaluation at three, six, and nine months by KSS and VAS score.

In group II, patients were given UC-II peptide 40 mg capsules along with NSAIDs and calcium 500 mg tablets. The suggested dose of the UC-II was one capsule a day or a minimum period of three months. Physical therapy in the form of fomentation and quadriceps exercises was continued throughout the treatment period. Follow-up evaluation at three, six, and nine months was done by the objective KSS and VAS score.

Data analysis

Between-group statistical testing was not performed due to the non-randomized, preference-based allocation, which may introduce confounding. Within-group changes were analyzed using paired t-tests, and between-group differences were described descriptively. Baseline characteristics, including age, sex, height, weight, and body mass index (BMI), were compared between the two groups at enrollment to confirm demographic comparability. No statistically significant differences were observed (all p > 0.05), ensuring that both cohorts were comparable at baseline. Digital X-rays were taken at every visit to assess the radiological effects of UC-II and the improvement in the mechanical axis in the operated case of HTO. Power analysis was performed post-hoc (target = 0.8, α = 0.05) and reported. Between-group adjusted analyses using ANCOVA and linear mixed-effects models (group×time) were added; results with 95% CIs and Cohen’s d were now included. Multiplicity control by Bonferroni correction is noted. 

## Results

A total of 54 patients were included, 24 male (45%) and 30 female (55%) patients, with a mean age of 52.7 ± 4.9 years. The largest age group was 51-55 years (21 patients; 39%), followed by 56-60 years (20 patients; 37%). According to the Kellgren-Lawrence (K-L) grading, most patients were Grade III (25; 46.3%), followed by Grade II (20; 37%) and Grade I (9; 16.7%). Baseline comparison showed no statistically significant difference between groups in age, sex, height, weight, or BMI (all p > 0.05), confirming demographic comparability.

In the HTO group, the mean KSS increased from 48.3 ± 6.9 preoperatively to 84.3 ± 7.1 at nine months, an absolute gain of +36.0 points (95%CI 31.9 to 40.1) (p < 0.001, paired t-test) (Table 1). The serial improvements were 63.3 ± 6.0 at three months, 75.7 ± 7.5 at six months, and 84.3 ± 7.1 at nine months; each interval comparison was significant (p < 0.001) (Table 2). In the HTO group, mean VAS pain scores decreased from 7.7 ± 1.2 preoperatively to 2.4 ± 0.9 at nine months, an absolute reduction of −5.3 points (95%CI −5.8 to −4.7) (p < 0.001) (Table 1). Gradual reduction was observed at each follow-up: 5.4 ± 0.9 at three months, 4.0 ± 0.8 at six months, and 2.4 ± 0.9 at nine months (Table 2).

**Table 1:** Mean KSS and VAS score of patients in both the treatment groups at baseline and at the nine-month follow-up. KSS: Knee Society Score; VAS: visual analog scale

Parameter	Time Point	Surgical Treatment (HTO), mean±SD	Absolute Change, Δ (95% CI)	p-value	Conservative Treatment, mean±SD	Absolute Change, Δ (95% CI)	p-value
Knee Society Score (KSS)	Baseline	48.3 ± 6.9			53.0 ± 6.7		
	9 months	84.3 ± 7.1	+36.0 (+31.9 to +40.1)	< 0.001	75.7 ± 9.0	+22.7 (+18.4 to +26.9)	< 0.001
Visual Analogue Scale (VAS)	Baseline	7.7 ± 1.2			7.0 ± 1.2		
	9 months	2.4 ± 0.9	−5.3 (−5.8 to −4.7)	< 0.001	3.1 ± 1.1	−3.9 (−4.5 to −3.3)	< 0.001

**Table 2:** Mean KSS and VAS score of patients undergoing high tibial osteotomy at the three-, six- and nine-month follow-up. KSS: Knee Society Score; VAS: visual analog scale

Score	Baseline	3-month follow-up, mean±SD	6-month follow-up, mean±SD	9-month follow-up, mean±SD	p-value
Knee Society Score	48.3±6.9	63.3±6.0	75.7±7.5	84.3±7.1	0.001
VAS for pain	7.7± 1.2	5.4 ± 0.9	4.0 ± 0.8	2.4 ± 0.9	0.001

In the conservative group, the mean KSS improved from 53.0 ± 6.7 at baseline to 75.7 ± 9.0 at nine months, giving an absolute increase of +22.7 points (95%CI +18.4 to +26.9) (p < 0.001) (Table 1). Progressive gains were 59.8 ± 7.7 at three months, 67.5 ± 8.8 at six months, and 75.7 ± 9.0 at 9 months; all were statistically significant (p < 0.001) (Table 3). In the conservative group, mean VAS decreased from 7.0 ± 1.2 to 3.1 ± 1.1 after nine months, an absolute decrease of −3.9 points (95% CI −4.5 to −3.3) (p < 0.001) (Table 1). The sequential means were 5.7 ± 1.5 at three months, 4.4 ± 1.3 at six months, and 3.1 ± 1.1 at nine months, all significant (p < 0.001) (Table 3).

**Table 3:** KSS and VAS score of patients undergoing conservative treatment at three-, six- and nine-months follow-up. KSS: Knee Society Score; VAS: visual analog scale

Score	Baseline, mean±SD	3-month follow-up, mean±SD	6-month follow-up, mean±SD	9-month follow-up, mean±SD	p-value
Knee Society Score	53.0±6.7	59.8±7.7	67.5±8.8	75.7±9.0	0.001
Visual Analogue Scale (VAS) for pain	7.0± 1.2	5.7 ± 1.5	4.4 ± 1.3	3.1 ± 1.1	0.001

Before HTO, all 27 patients had a poor KSS. At nine months after HTO, none were poor, three fair, eight good, and 16 excellent (Table 4).

**Table 4:** KSS grading of patients undergoing high tibial osteotomy at baseline and the nine-month follow-up (N=27) KSS: Knee Society Score

KSS Grade	Baseline, n (%)	9-month follow-up, n (%)
Poor	27 (100%)	0
Fair	0	3 (11.1%)
Good	0	8 (29.7%)
Excellent	0	16 (59.2%)

Before conservative treatment, 22 patients had a poor KSS, and five had a fair KSS. At nine months of treatment, 12 were good, eight fair, one poor, and six excellent (Table 5).

**Table 5:** KSS grading of patients undergoing conservative treatment at baseline and at the nine-month follow-up (N=27) KSS: Knee Society Score

KSS Grade	Baseline, n (%)	9-month follow-up, n (%)
Poor	22 (81.5%)	1 (3.8%)
Fair	5 (18.5 %)	8 (30%)
Good	0	12 (44%)
Excellent	0	6 (22.2%)

Before HTO, 22 patients showed severe pain on the VAS scale, and five patients showed moderate pain. At nine months, 22 patients had mild pain, and five patients had moderate pain. Before conservative treatment, 19 patients showed severe pain on the VAS, and eight patients showed moderate pain. At nine months, 19 patients had mild pain, and eight patients had moderate pain. There was no patient with severe pain at nine months.

**Figure 1:**
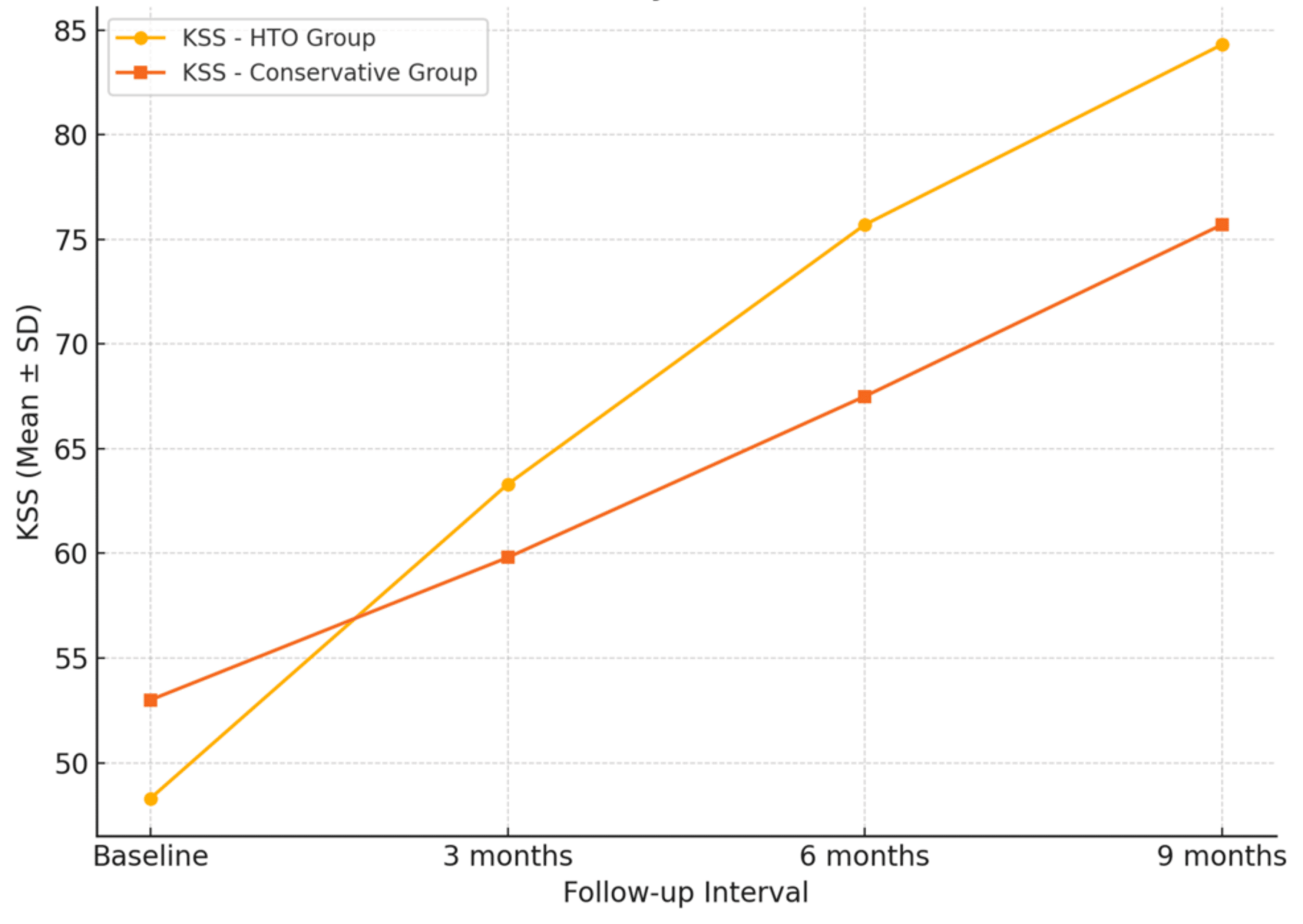
Trend of Knee Society Score (KSS) over time in surgical (HTO) and conservative treatment groups Both groups showed progressive improvement, with greater absolute gains in the HTO cohort.

**Figure 2:**
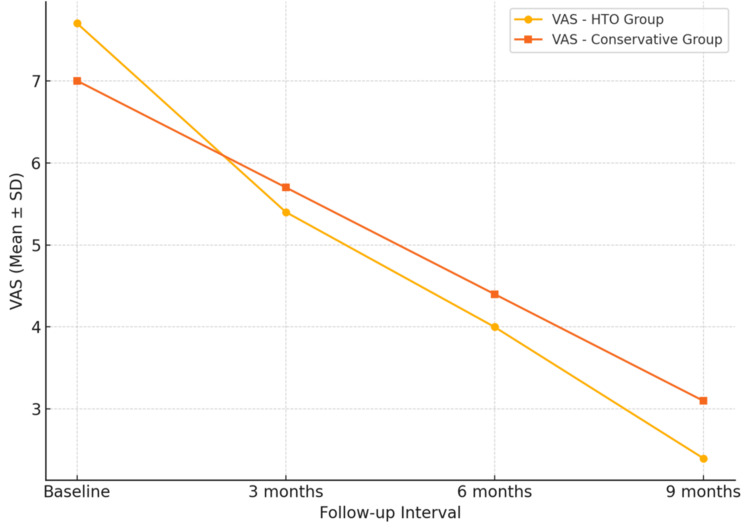
Trend of Visual Analogue Scale (VAS) for pain over time in surgical (HTO) and conservative treatment groups. Pain scores decreased significantly in both groups, with more marked reduction following HTO.

The progression of mean KSS and VAS values at baseline, three, six, and nine months for both treatment groups is illustrated in Figure 1 and Figure 2, respectively. These demonstrate consistent improvement in function and reduction in pain over time in both groups, with greater absolute gains observed in the HTO group. Although greater improvements in KSS and VAS were observed in the HTO group, these represent associative trends rather than statistically confirmed between-group differences.

## Discussion

KSS in patients* *undergoing* *HTO

In our study, the objective KSS of the patients undergoing HTO was 48.3±6.9 at the beginning of the study, which increased up to 84.3±7.1 postoperatively at the end of nine months. This improvement in KSS was statistically significant with a p-value of 0.001 by the paired samples t-test. Billings et al., in their study, performed HTO in 64 knees, of which 43 knees had good or excellent clinical results, with an average knee score of 94 points at an average of 8.5 years after the osteotomy, and 21 knees had subsequent total knee arthroplasty at an average of 65 months after the HTO [7]. In our study, the KSS after HTO improved from 63.3±6.0 at three months, 75.7±7.5 at six months, and 84.3±7.1 at nine months. Multiple pairwise comparisons of the mean KS scores between different time intervals were also statistically significant with a p-value of 0.001.

Similar findings were seen in the study by Ballal et al., where the mean KSS improved significantly from 98.83 ± 10.78 preoperatively to 185.67 ± 11.12 at the 12-month follow-up (P < 0.001) [8]. The KSS showed gradual improvement postoperatively, measuring 134.50 ± 7.47 at three months and 168.33 ± 11.17 at six months. In a study conducted by Evgenievich et al., comparable enhancements were noted in the objective score of the KSS [9]. Before surgery, the average objective score was 43.66 ± 11.50 points. Three months after surgery, there was a notable improvement in the objective score to 77.72 ± 9.48, accompanied by reduced pain intensity. By one year postoperatively, the scores further increased to 81.93 ± 6.65.

VAS for pain in patients undergoing HTO

In our study, the mean VAS for pain score was 7.7± 1.2 before HTO. After nine months from the HTO, the mean VAS score reduced to 2.4 ± 0.9. This improvement in VAS score was statistically significant with a p-value of 0.001 by paired samples t-test. The improvement in VAS score after HTO was seen as 5.4 ± 0.9 at three months, 4.0 ± 0.8 at six months, and 2.4 ± 0.9 at nine months. In the study by Van Outeren et al., the estimated mean VAS score at the 12-month follow-up in the patients of the usual care treatment group was 5.3, while for the HTO group, it was 3.6, resulting in a mean difference of 1.7 [10]. Mukherjee et al., in their study, showed that the VAS score improved from 5.77 to 1.5 at the six-month follow-up in 42 HTO patients [11].

KSS in patients undergoing conservative treatment

In our study, the patients receiving conservative treatment had a mean KSS of 53.0±6.7 at the beginning of treatment. After nine months, the mean KSS had increased to 75.7+9.0. This improvement in KSS was statistically significant with a p-value of 0.001 by paired samples t-test. The KSS increased gradually from 53.0±6.7 preoperatively to 59.8±7.7 at three months, 67.5±8.8 at six months, and 75.7±9.0 at nine months postoperatively. However, our study is one of the few studies done that evaluated improvements in knee OA by UC-II and other conservative drug treatments like NSAIDS in terms of KSS at three, six, and nine months of treatment.

VAS score in patients undergoing conservative treatment

In our study, the 27 patients receiving conservative treatment had a VAS score of 7.0± 1.2 at the beginning, which improved to 3.1+1.1 at the end of nine months (p-value -0.001). The VAS improvement was gradual from a baseline of 7.0± 1.2 to 5.7 ± 1.5 at three months, 4.4 ± 1.3 at six months, and 3.1 ± 1.1 at nine months. Sadigursky et al. had 53 patients in the UC-II treatment group, and their baseline VAS score was 7.1 ± 0.9; at the one-month follow-up, the VAS score reduced to 5.1 ± 1.3, and at the three-month follow-up, the VAS score further reduced to 3.4 ± 1.6 [12]. This reduction in VAS score was statistically significant with a p-value of < 0.001. In the study by Oentarini et al., the VAS score significantly decreased by 67.9% in subjects who received UC-II supplementation, whereas the placebo group only showed a reduction of 12.2% [13].

KSS grade of outcome in patients undergoing HTO

The objective KSS of <60 points is considered as poor grade, 60-69 points as fair, 70-85 points as good, and 85-100 points as excellent. In our study, all 27 patients (100%) before HTO had a poor KSS grade. At nine months, no patient was in the poor grade, three patients (11.1%) were in fair grade, and eight patients (29.7%) were in good KSS grade. An excellent grade was reported in 16 patients (59.2%) at the end of nine months postoperatively. In a study by Manoharan et al., the KSS grade outcome was excellent in 15%, good in 65%, fair in 15% and poor in 5% after a mean follow-up of 20 months in HTO patients [14].

KSS grade of outcome in patients undergoing conservative treatment

Before the start of conservative treatment in 27 patients in our study, 22 patients belonged to the poor KSS grade, and five patients belonged to the fair KSS grade. At the nine-month follow-up, 12 patients belonged to a good KSS grade, followed by eight patients in a fair grade. An excellent grade was shown by six patients, and one patient was still in the poor grade. However, no comparative study is available for KSS evaluation in patients receiving UC-II for early knee OA.

VAS grading of pain after HTO and after conservative treatment

The severity of pain according to VAS grading in our study was classified as no pain (0 points), mild pain (1-3 points), moderate pain (4-6 points), severe pain (7-9 points), and very severe pain (10 points) [15]. Before HTO, 22 (81.5%) out of 27 patients reported severe pain, and five (18.5%) patients reported moderate pain. Nine months after surgery, 22 (81.5%) patients had mild pain, and only five (18.5%) reported moderate pain. In our study, before the start of the conservative treatment, 19 patients reported severe pain, and eight reported moderate pain. At the nine-month follow-up, 19 patients reported mild pain, and only eight reported moderate pain.

Limitations and future directions

Conservative treatment and HTO are both viable options for the treatment of OA in young active patients. However, as HTO shifts the weight-bearing axis to the lateral side, it relieves symptoms and delays the need for total knee arthroplasty for the long term. 

Nevertheless, the study’s methodological limitations warrant careful interpretation. Allocation to treatment groups was based on patient preference, introducing potential selection bias and confounding by indication. The design was non-randomized, and no propensity-based adjustment (e.g., matching or IPTW) could be performed given the modest sample size, leaving room for unmeasured confounders. Outcome assessors were not blinded to treatment allocation, which may have introduced measurement bias, especially since both KSS and VAS are subjective measures.

Furthermore, as between-group adjusted analyses were not performed, these results should be interpreted as associational rather than causal. The comparative trends observed likely reflect the mechanical correction and load redistribution achieved by osteotomy, whereas conservative therapy may have provided symptomatic benefit through biochemical modulation. Because the study was non-randomized, lacked intergroup statistical analysis, and included concomitant NSAID use in both groups, the independent effect of collagen supplementation cannot be determined. These findings should therefore be interpreted as associational, not causal. Larger randomized studies with clearly separated treatment arms are needed to confirm the comparative and independent effects of these interventions

The study was also limited by a short-term follow-up of nine months, which restricts assessment of long-term outcomes such as durability of improvement, radiographic progression, and time to arthroplasty. Additionally, systematic adverse event reporting was not performed and should be incorporated in future studies to better assess treatment safety profiles. Finally, the generalizability of these results is limited to active, middle-aged patients with mild-to-moderate varus deformity (<20° correction) and early-to-moderate medial compartment OA. Findings may not apply to patients with severe deformity, multi-compartmental disease, or systemic comorbidities.

Despite these limitations, the results suggest that HTO remains a valuable joint-preserving option for selected patients with early medial knee OA, providing meaningful short-term functional gains and pain relief. Conservative management with UC-II and NSAIDs also showed significant symptomatic improvement and may be considered a viable non-surgical alternative in appropriately selected cases. Future randomized controlled studies comparing collagen-only, NSAID-only, and combination therapy arms are needed to establish the independent efficacy of UC-II in early OA. 

## Conclusions

This study evaluated the short-term clinical outcomes of HTO compared with conservative management using UC-II in active middle-aged adults with early medial compartment OA of the knee. Both treatment strategies were associated with significant improvements in pain and function as reflected by increases in the KSS and reductions in the VAS score over a nine-month follow-up period. HTO was associated with greater short-term improvement in both functional and pain outcomes compared to conservative management. Both HTO and conservative management with UC-II plus NSAIDs were associated with significant short-term improvements in pain and function among active middle-aged adults with early medial knee osteoarthritis.

The consistent short-term improvement in both groups highlights that joint-preserving interventions, whether surgical or conservative, can play an important role in the early stages of unicompartmental disease. Future studies incorporating randomized or propensity-matched designs, blinded assessments, long-term follow-up, and radiological evaluation are warranted to validate these findings and define the comparative effectiveness and durability of surgical versus conservative interventions in early OA of the knee.
